# Comparative analysis of the effect of autoclaving and 10% formalin storage on extracted teeth: A microleakage evaluation

**DOI:** 10.4103/0972-0707.53338

**Published:** 2009

**Authors:** Kanika Attam, Sangeeta Talwar, Seema Yadav, Sanjay Miglani

**Affiliations:** Department of Conservative Dentistry, Maulana Azad Institute of Dental Sciences, University of Delhi, New Delhi - 110 002, India

**Keywords:** Autoclaving, formalin, microleakage

## Abstract

**Aim:**

This study compares the effect of formalin and autoclaving the tooth samples by evaluating microleakage *in-vitro*.

**Materials and Methods:**

Forty-five extracted human permanent incisor teeth were taken and randomly divided into three groups (with different methods of storage and disinfection) with 15 teeth each: Group 1: Control-extracted teeth in this group were stored in normal saline, Group 2: the extracted teeth in this group were stored in 10 % formalin for two weeks and Group 3: the extracted teeth were Autoclaved at 121°C, at 15 psi pressure for 40 minutes. In all the groups after the specified storage period, class V cavities were prepared on the labial surface and restoration was performed with Z100 restorative. Finished and polished samples were subjected to 500 cycles of thermocycling. All specimens were immersed in methylene blue for 24 hours. After sectioning, the margins of restoration were evaluated for dye leakage at 10 X magnification, using an optical microscope. Data were subjected to nonparametric Kruskal Wallis one way analysis of variance. Inter-group comparisons were performed using the Mann Whitney test (*P* < 0.05).

**Results:**

The authors found that the microleakage in the formalin group was considerably lower than that in the control group. The autoclave group showed slightly higher mean microleakage, but the difference was not statistically significant.

**Conclusion:**

Both autoclaving and formalin storage affect, to a varying degree, the microleakage values *in vitro*. The results in the autoclaving group matched those of the control group more closely, with only a slight difference.

## INTRODUCTION

Extracted human or bovine teeth are frequently used for preclinical work, research on microleakage detection, and for dentin bond strength and hardness tests for novel products. These teeth are obtained from a variety of sources like animal houses, cadavers, and so on. The pulp and periradicular tissues of these extracted teeth are loaded with a number of viable bacteria and their toxins, including Hepatitis B virus (HBV) and Human immunodeficiency virus (HIV), which may act as potential sources of infectious disease.[1]Furthermore, tooth preparation in the technique laboratory is generally done without a liquid coolant. Certainly then the risk exists for the spread of pathogen through both aerosol and accidental puncture wounds. Therefore, it is crucial that a medium is used to clean and disinfect the extracted teeth.

There are various methods available for sterilization / disinfection of extracted teeth, such as, Chloramine, Formalin, Sodium hypochlorite, Thymol, Alcohol, Antibiotics, Glutaraldehyde, Autoclaving, Dry heat, Distilled water, Normal saline or Freezing, 1:10 Household bleach, Ethylene oxide sterilization, and Gamma radiation. Ethylene oxide has been found to be only 20 – 36 % effective against *B. subtilis *spores in extracted teeth.[2]

J. M. White conducted an experiment on sterilization of extracted teeth and compared gamma irradiation with autoclaving, ethylene oxide, and dry heat. He concluded that Gamma radiation sterilizes teeth and endodontic filling material without altering the structure and function of dentin. A dose of 173 krad, with the help of the Cesium radiation source, was required for complete sterilization.[3] However, Gamma radiation requires expensive equipment, which is not readily available. Chemicals like 2.6% sodium hypochlorite, 3% hydrogen peroxide, and boiling in water are not effective for disinfecting teeth.[4]

Dominici *et al.,* in their study, concluded that only autoclaving for 40 minutes at 240°F and 20 psi or soaking in 10% formalin for one week are 100% was effective in preventing the growth of * B. stearothermophilus * spores.[5] Iodophor, glutaraldehyde, synthetic phenol, and sodium hypochlorite failed to disinfect many of the teeth samples. Thus, these agents could not be relied upon. Glutaraldehyde appeared to be effective on the external surface of teeth, but did not penetrate to the pulp chamber.[1]

Directives by the American Dental Association (ADA) and the Center for Disease Control (CDC) call for a thorough removal of any organism capable of transmitting disease. The extracted teeth should be cleaned of visible blood and gross debris and maintained in a hydrated state in a well-constructed, closed container, during transport. The container should be labeled with the biohazard symbol. Before being used for educational or research purposes the teeth samples should be stored in 10% formalin for two weeks or autoclaving for 40 minutes at 121° C and 15 psi pressure.[6]

Difficulties exist in the use of extracted teeth, as they are grossly contaminated, difficult to sterilize, and may be damaged or altered by the sterilization process.[7] There have been concerns regarding the effect of formalin storage of samples affecting the bond strength, microleakage, and dentin permeability. Whether autoclaving affects dentinal structure to the point where the micro-chemical relationship between dental materials and the dentin would be affected is unknown.[8]

Therefore this * in vitro* research was undertaken to evaluate the effect of formalin storage and autoclaving on extracted human teeth, to be used for microleakage evaluation.

## MATERIALS AND METHODS

Tooth material: Freshly extracted 45 human maxillary incisor teeth were obtained and kept in saline for 24 hours. Only teeth that were free of caries, cracks or previous restoration were selected. They were randomly divided into three groups of 15 teeth each.

Group 1: Control: The teeth were kept in saline throughout the procedure.

Group 2: The teeth were stored in 10% formalin (LOBA Chemie Pvt. Ltd., Mumbai, India) for two weeks.

Group 3: The extracted teeth were autoclaved (Euroklav 23 V-S, Melag, Germany) at 121°C and 15 psi pressure for 40 minutes. After autoclaving the teeth were stored in sterile saline until use.

The teeth were then debrided of the soft tissue, calculus, and bone. Any handling of teeth was done by wearing gloves, mask, and protective eyewear. Class V cavities were prepared at the cementoenamel junction (CEJ) of the labial surface of all the specimens using number 245 carbide bur (SS White, USA). The depth of the cavities was kept at 0.5 mm into the dentin and the margins as butt joints. The preparation dimension of 1.5 mm (depth) × 1 mm (occlusogingivally) × 4 mm (mesiodistally) was kept constant for all the teeth. During the procedure the teeth were held in a piece of moistened gauze so as to keep them from getting brittle.

After cleaning and drying the cavity preparations, etching was performed with Scotchbond Multipurpose (3M ESPE dental products, St Paul MM, USA) for 15 seconds. The specimens were then rinsed and dried. A thin layer of adhesive resin, Adper Single Bond 2 (3M ESPE dental products, St Paul MM, USA) was applied and photocured according to the manufacturer's instructions. Restoration was performed using Z 100, B2 shade (3M ESPE dental products, St Paul MM, USA) restorative, and photocured using light-emitting diode (LED) curing light (Gnatus, Brazil), for 40 seconds. Finishing and polishing was performed using super snap disc (Shofu Inc. Japan), at slow speed (10,000 RPM) with no separate spray of water as per manufacturer′s instructions. The teeth were stored in saline at 37°C for 24 hours. The teeth were subjected to 500 cycles of thermocycling between 5 to 55°C with a dwell time of 30 seconds.

## Microleakage detection

The surfaces of the teeth were coated with two layers of nail varnish except that the restoration and 1 mm area around the restoration and apical foramen was blocked with modeling wax (MDM Corporation, India). The samples with the apices blocked were then immersed in 1% methylene blue dye (pH = 7) (S.D. Fine Chemical Ltd, India) for 24 hours,[9] to allow the dye to penetrate through the cavity margin (if any). The specimens were then washed and the varnish coating was stripped off using scalpel blade. Sample sectioning was performed bucco-lingually from the center of the restoration, using a slow speed diamond wheel under water cooling.

The samples were examined under a binocular microscope (Labomed CXR II, Ambala Cantt., India) at 10 X magnification. The depth of the dye penetration was scored[9] as shown in Table 1.

As every sample was sectioned, two readings were obtained from each sample. Therefore, a total of 30 readings were obtained for each group. The data was then subjected to statistical analysis.

## Statistical analysis

To determine a statistically significant difference in the quantity of leakage at the restoration margin, data were analyzed using nonparametric Kruskal Wallis one way analysis of variance. Inter-group comparisons were done using the Mann Whitney test. * P* < 0.05 was considered as significant. The statistical calculations were executed using Epi Info 2002 software.

## RESULTS

The following scores were obtained for all three groups [Table 2].

The mean scores for control, formalin, autoclave groups are 2.23, 1.40, and 2.33, respectively. The median scores for control, formalin, autoclave groups are 2.50, 1.00, and 2.00, respectively [Table 3]. There was a significant difference among the median scores (* P* = 0.01). The microleakage in the formalin group was significantly lower than that in the control group (* P* = 0.02). The median microleakage in the autoclave group was higher than that in the control group, but not statistically significant (* P* = 0.96). The median microleakage difference between the autoclaving and formalin groups was very high, showing almost no correlation in the result (* P* = 0.01).

## DISCUSSION

Disinfection/sterilization are crucial steps that are to be performed before handling extracted teeth. It is important that the storage media that provides complete sterilization does not affect the properties of the tooth, does not interfere with the test planned subsequent to sterilization, and does not affect the “feel” and cutting characteristics of the teeth.

**Table 1:** Score for dye penetration

Score	Criteria
0	No evidence of dye penetration
1	Dye penetration along cavity wall up to one-third the cavity depth
2	Penetration greater than one-third, but less than two-thirds of cavity depth
3	Penetration greater than two-thirds of cavity depth, but not along the dentinal tubules
4	Penetration to cavity depth, and along the dentinal tubules

**Table 2:** Microleakage distribution scores

Group	Number of redings	Score 0	Score 1	Score 2	Score 3	Score 4
Control	30	4*	3	8	12	3
		(13.3)	(10.0)	(26.7)	(40.0)	(10.0)
Formalin	30	11	7	4	5	3
		(36.7)	(23.3)	(13.3)	(16.7)	(10.0)
Autoclave	30	1	5	11	9	4
		(33)	(16.7)	(36.7)	(30.0)	(13.3)

* Number of teeth in the corresponding group showing the corresponding score, figures in parentheses are in percentages

**Table 3:** Mean, median, 25, 75 percentile values of microleakage scores

	Control	Formalin	Autoclave
Mean	2.23	1.40	2.33
Median	2.50	1.00**	2.00++
Percentile			
25	1.75	0.00	2.00
75	3.00	3.00	3.00

** p = 0.02 significant difference between formalin and control group

++ p = 0.01 significant difference between formalin and autoclave group

Formalin, an electrophilic agent, is composed of formaldehyde, methyl alcohol, and sodium acetate in water. It preserves tissues by cross-linking proteins, glycoproteins, nucleic acid, and polysaccharides to form insoluble methylene bridge products.[10] Formalin in 10% concentration is the only disinfectant solution that penetrates the pulp chamber of teeth in what would be considered as an effective antimicrobial concentration. A minimum exposure time of two weeks is required for a complete disinfection of teeth.[1] Although formalin is the most effective disinfectant; it is a hazardous material and a potential carcinogen. Therefore, the container holding the teeth must be opened only under a fume hood and the health care personnel must use protective personal equipment.[11,12]

## Autoclaving

Pantera and Schuster reported that autoclaving extracted teeth for 40 minutes (20 minutes is inadequate) at 121°C and 15 psi in either saline or autoclave bags renders them free of viable microorganisms.[7] Although autoclaving of teeth may cause a slight reduction in dentin microhardness, it does not alter the physical properties sufficiently to compromise on the strength * in vitro.*[7,8] However, extracted teeth with amalgam restorations cannot be autoclaved because of the mercury vapor released in the air. Therefore, they must only be treated with 10% formalin.

Both formalin and autoclaving treatments are simple, inexpensive, and suitable for routine use, do not alter the “feel” and cutting characteristics of the teeth, and effectively destroy all kinds of microorganisms, including viruses. The results of this study show that the microleakage value may be altered by the sterilization process used. This is in accordance with other studies. Haller evaluated the effect of formalin on class V restoration microleakage and found a decrease of over four weeks.[13]

Wieczkowski evaluated the effect of formalin on bond strength and found a decrease in dentin bond strength after immersion in formalin for one year.[14] George SW * et al.,* demonstrated that formalin storage of samples resulted in a significant decrease in apical microleakage compared to the nonfixed samples. The mechanism leading to a decrease is uncertain, but may be related to an alteration of dentinal tissues by fixing the properties of formalin. Storage in formalin may have promoted the crosslinking of collagenous proteins present in the pulp and dentin matrix, leading to an accumulation of insoluble products along the canal wall after instrumentation.[15] A formalin impregnated smear layer may have formed during cavity preparation. This layer seems to be resistant to the etching effect of phosphoric acid. Michael Pichardo * et al.* postulated that stabilization of the collagen may prevent their collapse, allowing increased mechanical interlocking of the restorative material to the dentin.[16] He also found a significant decrease in the microleakage in teeth stored in formalin when compared to freshly extracted teeth.

Autoclaving has no effect on bond strength and dentin permeability studies.[17] Jason Jonghyuk Lee in his study commented that autoclaving negatively affected the shear bond strength values of specimens initially stored in distilled water. He concluded that storage and simultaneous sterilization in 10% formalin was the most logical treatment for the extracted teeth that will be used in the dentin bonding research.[18]

Microleakage can be defined as a clinically undetectable passage of bacteria, fluids, molecules or ions between a cavity wall and a restorative material applied to it. Detection of microleakage can be accomplished by a number of techniques, including, bacteria, chemical or radioactive tracer molecule, fluid permeability, and dye penetration. Although no method used for the detection of microleakage is ideal, dye penetration is regarded as the most practical method, with an accepted degree of reliability.[19] This method allows the production of sections showing leakage, in contrasting colors, to both tooth and restoration, without the need for further chemical reaction or exposure to potentially hazardous radiation. The limitations of dye penetration include subjectivity and sample destruction.

This is the first study evaluating the effect of autoclaving on microleakage. In this study it is found that the autoclaved samples show a slight increase in microleakage, although not statistically significant. Disruption of the collagen molecule by heat and pressure can affect the ionic bond between the collagen and hydroxyapatite, while the molecular structure of the collagen remains unaffected. This can alter the hybrid layer formed after resin penetration and thus increase the microleakage value detected * in vitro* .[8]

## CONCLUSION

Within the limitation of this study it may be concluded that: 1) Both autoclaving and formalin storage affect, to a varying degree, the microleakage values * in vitro* . Formalin storage of the samples results in significantly decreased microleakage detection scores. 2) The results in the autoclaving group matched those of the control more closely, with only a slight difference. Therefore, of the available media, autoclaving gives the best results. 3) There exists a need for more research, to develop a media that sterilizes without affecting the properties, so as to match the * in vivo* conditions more accurately.

## ACKNOWLEDGMENT

The authors would like to thank Dr. J. Augustine for his help and support in the microscopic analysis of the samples and Mrs. Renuka Saha for the statistical evaluation of the data.
